# Right on TARGET: glutamine metabolism in cancer

**DOI:** 10.18632/oncoscience.205

**Published:** 2015-08-20

**Authors:** Boris Ratnikov, Young Joo Jeon, Jeffrey W. Smith, Ze'ev A. Ronai

**Affiliations:** ^1^ Cancer Center, Sanford Burnham Prebys Medical Discovery Institute, La Jolla, CA, USA

**Keywords:** glutamine, metabolism, cancer, SLC1A5, RNF5

## Abstract

Recent studies highlight the importance of glutamine metabolism in metabolic reprogramming, which underlies cancer cell addiction to glutamine. Examples for the dependence on glutamine metabolism are seen across different tumor types as during different phases of cancer development, progression and response to therapy. In this perspective, we assess the possibility of targeting glutamine metabolism as a therapeutic modality for cancer.

## INTRODUCTION

Oncogenic transformation leads to increased cell proliferation that increases the requirement for nutrients and energy, which triggers metabolic reprogramming [1]. Addiction to glutamine is part of the reprogramming process and is a frequent hallmark of cancer cells. Consequently, glutamine deprivation often induces tumor cell death [2].

**Figure 1:**
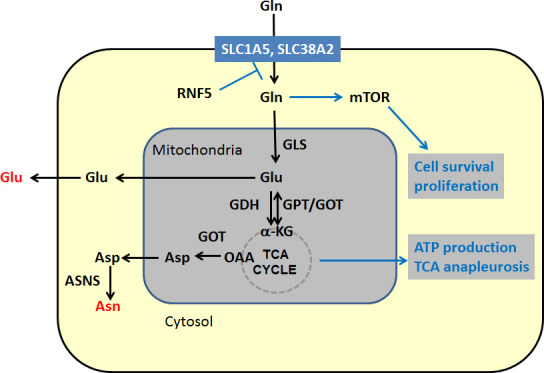
Schematic representation of key steps in glutamine metabolism Black arrows denote chemical reactions and blue lines other effects, such as activation and down regulation. Key outputs of glutamine metabolism are shown in red. Abbreviations: GLS – kidney type Glutaminase, GDH – glutamate dehydrogenase. GPT – alanine aminotransferase, GOT – aspartate aminotransferase, ASNS – asparagine synthetase, α-KG – alpha ketoglutarate, OAA-oxaloacetate.

Glutamine, the most abundant amino acid in blood [3], is a major donor of nitrogen. In cancer cells it becomes a carbon source [4, 5, 6, 7, 8] to support TCA anapleurosis and biosynthetic reactions, which are required to maintain proliferative phenotype (Figure 1). Key components in glutamine contribution to central carbon metabolism include solute carrier proteins (i.e., SLC1, 5, 6, 7, 35, 38) and others [9], which are responsible for uptake of glutamine into the cell, and glutaminase (GLS), an enzyme that converts glutamine to glutamate in mitochondria, thus driving glutamine utilization in the TCA cycle.

Expression of glutamine carrier proteins directly determines levels of intracellular glutamine, which is exchanged (via SLC7A5) with leucine, affecting mTORC1 activity. Upon its conversion to glutamate by GLS, glutamine provides a key source of carbon for the TCA cycle, [9]. Many glutamine transporters and GLS are upregulated in cancer [10].

Elevated uptake and availability of glutamine in cancer cells can be attributed to both transcriptional and post translational regulation. Transcriptionally, c-Myc, which is often deregulated in cancer, induces the transcription of glutamine transporters SLC38A5 and SLC1A5 and upregulates expression of GLS, via its silencing of mir23a/b expression [11]. Via ATF4, Myc also induces apoptosis upon glutamine deprivation [12, 13], which can be attenuated by asparagine – a product of both glutamine carbon and nitrogen metabolism [14]. K-Ras contributes to glutamine utilization pathway by rerouting glutamine carbon flow into the TCA cycle, through downregulating expression of GLUD1 (GDH) and activation of GOT1 [15]. Glutamine anaplerosis is also regulated by SIRT4 [16] and antagonized by mTORC1 mediated activation of glutamate dehydrogenase (GDH) [17].

In our recent report [18], we identified a new way in which glutamine metabolism is regulated. The microtubule-targeting chemotherapeutic reagent paclitaxel triggers endoplasmic reticulum (ER) stress, thereby promoting proteosomal degradation of two glutamine transporters SLC1A5 and SLC38A2 (Figure 1). This degradation is specifically mediated by the ubiquitin ligase RNF5 and ultimately leads to mTOR inactivation, autophagy and apoptosis of breast cancer cells [18]. RNF5 regulates the turnover of SLC1A5 and SLC38A2 in about 30% of breast cancer patients that are responsive to taxanes-based therapies, which is associated with better prognosis. Overall, the level of the glutamine carrier proteins SLC1A5 and SLC38A2 was found to be excellent predictor of the outcome for breast cancer patients where a low level of these carrier proteins associates with a better outcome, including a better response to therapy [18]. The latter suggests that attenuating either the expression or the activity of SLC1A5 could render tumors more susceptible to therapy. Likewise, the inhibition of glutamine metabolism by targeting different steps along this metabolic pathway is expected to attenuate tumor growth.

Among the questions that remain to be addressed is the importance of glutamine metabolism in the tumor microenvironment, as opposed to the tumor per se. The dynamic interactions between the cancer-associated stromal cells (i.e., fibroblasts, endothelial and immune cells), and the tumor are key drivers of malignancy. Expression and activity of glutamine carrier proteins in stromal cells is expected to differ from the core tumor due to a different oxygen tension as well as altered transcriptional and post translational control mechanisms. At present the impact of altered glutamine metabolism in stroma, and its effect on tumor progression and response to therapy, remains an important area that deserves careful assessment.

These observations support the idea that inhibitors of glutamine metabolism should be evaluated for clinical impact in oncology. A small molecule inhibitor of glutamine transport (gamma-l-glutamyl-p-nitroanilide) is currently being evaluated in clinical trials [19] and GLS has been validated as a therapeutic target in animal models of some cancers [20-22], leading to Phase I trials of the GLS inhibitor (CB-839) (https://www.clinicaltrials.gov/). Additional clinical implications include the potential use of aspects of glutamine metabolism to stratify patients for particular therapies or to monitor their effectiveness, as we have demonstrated for SLC1A5 [18]. A greater understanding of glutamine metabolism in both the tumor and in its microenvironment (i.e., stroma, immune system) will enable assessment of opportunities for the fine-tuning of this important regulatory axis as a way to antagonize uncontrolled cell growth.
